# Tuberculous Pancreatic Abscess: A Rare Condition Mimicking Carcinoma

**DOI:** 10.1155/1992/79652

**Published:** 1992

**Authors:** P. Watanapa, V. Vathanopas

**Affiliations:** 1 Department of Surgery Siriraj Hospital Mahidol University Bangkok 10700 Thailand; 2 Department of Surgery Hammersmith Hospital RPMS Du Cane Road London W12 ONN United Kingdom

## Abstract

We report a rare case of obstructive jaundice caused by a tuberculous abscess in the head of pancreas, mimicking carcinoma. The case was successfully treated by pylorus-preserving proximal pancreatoduodenectomy and antituberculous drugs.


*HPB Surgery*, 1992, Vol. 5, pp. 209–213
Reprints available directly from the publisher
Photocopying permitted by license only

© 1992 Harwood Academic Publishers GmbH
Printed in the United Kingdom

### CASE REPORT

### TUBERCULOUS PANCREATIC ABSCESS: A RARE CONDITION MIMICKING CARCINOMA

P. WATANAPA and V. VATHANOPAS
*Department of Surgery, Siriraj Hospital, Mahidol University, Bangkok 10700, Thailand*

*(Received 8 August 1991)*

We report a rare case of obstructive jaundice caused by a tuberculous abscess in the head of pancreas, mimicking carcinoma. The case was successfully treated by pylorus-preserving proximal pancreatoduodenectomy and antituberculous drugs.

KEY WORDS: Obstructive jaundice, tuberculosis, pancreas

### CASE REPORT

A 26-year-old man presented with painless obstructive jaundice. He gave a two-month history of malaise, low grade fever, anorexia, pale stools, increasing pruritus and 8 kg. weight loss. There was no history of gallstone disease or previous jaundice.

On admission he was deeply jaundiced with no palpable abdominal mass. Investigation confirmed an obstructive jaundice with marked elevation in serum bilirubin (33.4 mg/dl, normal 0.2 - 1.0 mg/dl) and alkaline phosphatase (191.0 Bodansky unit/L, normal 1.5 - 4.0 unit/L). Serum albumin was 34 g/L and transaminase levels were slightly raised. Chest radiography showed a fibrotic lesion at the right apex suggestive of old pulmonary tuberculosis (despite a negative history). Sputum examinations for acid-fast bacilli were negative on three consecutive days. Abdominal ultrasonography showed a distended gallbladder with a dilated common bile duct (2.0 cm diameter) and intrahepatic ducts. No gallstones were seen, but the pancreas was obscured by gas in the stomach. Cholangiography was not obtained.

At laparotomy a large, hard and irregular mass (circa 5 cm diameter) was found in the head of pancreas with dilatation of the biliary tree. There were a few small lymph nodes (0.5-1.0 cm in diameter) along the superior border of the pancreas and mesenteric root; frozen section revealed no evidence of malignancy. Carcinoma of the head of pancreas was diagnosed and because trial dissection was favourable

Address correspondence to Dr P. Watanapa, Department of Surgery, Hammersmith Hospital, RPMS, Du Cane Road, London W12 ONN, United Kingdom pylorus-preserving proximal pancreatoduodenectomy was performed. Gross examination of the specimen revealed a cavity in the pancreatic head, 2.5 cm in diameter and containing caseous material. Histopathology revealed tuberculosis of the pancreas with caseation but no acid-fast bacilli (Figure 1). Postoperative period was uneventful with removal of the nasogastric tube at 8 days. Antituberculous treatment with isoniazid and ethambutal was given for 18 months, together with initial three months of streptomycin injection. Two years after operation the patient is well and free of symptoms.

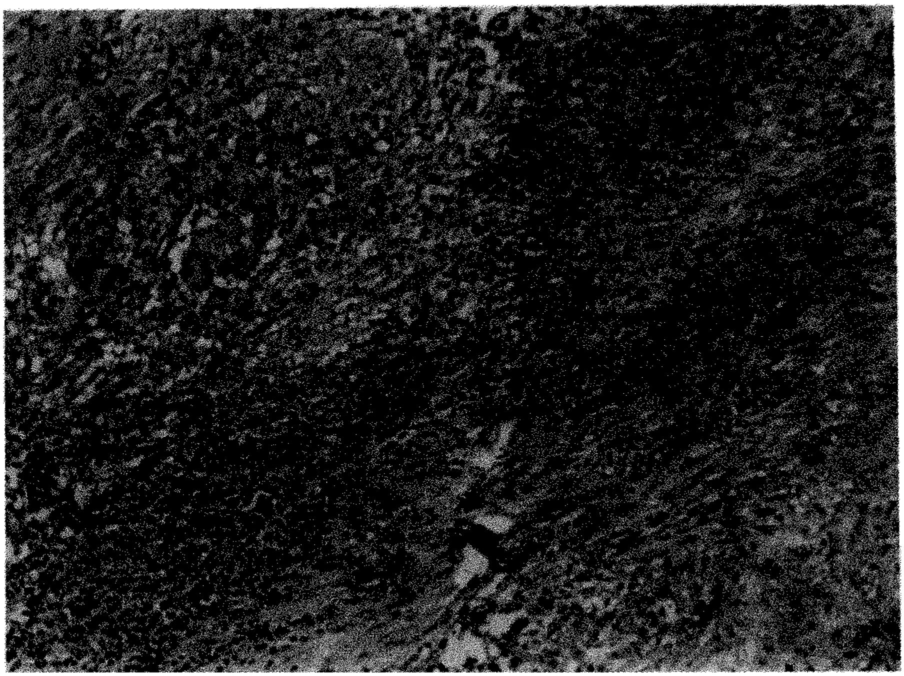

**Figure 1** Histopathology showed multiple small round cells infiltration with large epitheloid and multinucleated giant cells. There was area of caseation but no acid-fast bacilli.

### DISCUSSION

Abdominal tuberculosis is a continuing problem in developing countries[1]. Although the disease is uncommon in the West, there have been occasional case reports of tuberculous infection of abdominal viscera, especially among immigrants to the United States or the United Kingdom[2,3]. Tuberculosis of the pancreas is rare and usually occurs as a part of disseminated disease[2,7–9,11,13,14]. Previous autopsy series of miliary tuberculosis gave incidences of 4.7% (14 of 297 cases) and 2% (11 of 526 cases) for pancreatic involvement[4,5]. Even in India, with a high prevalence of tuberculous infection, Bhansali did not find a single case of pancreatic involvement in a review of 300 cases of abdominal tuberculosis[6].

Tuberculosis of the pancreas presents in several ways: acute pancreatitis[7], chronic pancreatitis[8], Trousseau's sign with a pancreatic mass mimicking carcinoma[9], obstructive jaundice (as in our case)[10], gastrointestinal bleeding[11] and chronic pyrexia with pancreatic abscess[2,12,13]. A mass in the pancreas found in patients with disseminated tuberculous infection should raise the possibility of pancreatic tuberculosis. In the present case, however, the lesion in the right lung looked inactive and there was no evidence of systemic tuberculosis. The weight loss plus the finding of a hard and irregular mass at the pancreatic head led us to make the diagnosis of adenocarcinoma of the pancreas. Perhaps CT scan would have shown a central cavity and needle biopsy obtained pus, but these investigations are not always available in a developing country.

Our case is the fourth case of tuberculous pancreatic abscess reported in the English literature. In the first case, the patient presented with persistent pyrexia, the diagnosis was only established after repeated laparotomies and the patient died[2]. In the second case, the diagnosis was made after laparotomy; the abscess was incised and drained and the patient improved rapidly with antituberculous treatment[12]. In the third case, pancreatic abscess was diagnosed without operative laparotomy in a patient with miliary tuberculosis. The abscess was aspirated under ultrasound guidance and the pus showed acid-fast bacilli. The patient responded well to antituberculous drugs.

The pathogenesis of pancreatic tuberculosis is still uncertain. Most cases arise secondary to tuberculous infection elsewhere and are associated with miliary or pulmonary tuberculosis. Perhaps mycobacteria reach the pancreas by lymphatic or haematogenous spread from the lung after reactivation of the pulmonary focus.

### *References*

1. Kapoor, V.K. and Sharma, L.K. (1988) Abdominal tuberculosis. *Br.J.Surg.*, **75**, 2–3
2. Stambler, J.B., Klibaner, M.I., Bliss, C.M. and Lamont, J.P. (1982) Tuberculous abscess of the pancreas. *Gastroenterology*, **83**, 922–925
3. Addison, N.V. (1983) Abdominal tuberculosis - a disease revived. *Ann.R.Coll.Surg.Engl.*, **65**, 105–111
4. Auerbach, O. (1944) Acute generalized miliary tuberculosis. *Am.J.Pathol.*, **20**, 121–136
5. Paraf, A., Menager, C. and Texier, J. (1966) La tuberculose du pancreas et la tuberculose des ganglions de l'etage superieur de l'abdomen. *Rev.Med.Chir.Mal.Foie*, **41**, 101–126
6. Bhansali, S.K. (1977) Abdominal tuberculosis. Experience with 300 cases. *Am.J.Gastroenterol.*, **67**, 324–337
7. Rushing, J.L., Hanna, C.L. and Selecky, P.A. (1978) Pancreatitis as the presentating manifestation of miliary tuberculosis. *West J.Med.*, **129**, 432–436
8. Stock, K., Riemann, J.F., Stadler, W. *et al.* (1981) Tuberculosis of the pancreas. *Endoscopy*, **13**, 178–180
9. Chandrasekhara, K.L., Iyer, S.K., Stanek, A.E. *et al.* (1985) Pancreatic tuberculosis mimicking carcinoma. *Gastrointes.Endosc.*, **31**, 386–388
10. Crowson, M.C., Perry, M. and Burden, E. (1984) Tuberculosis of the pancreas: a rare cause of obstructive jaundice. *Br.J.Surg.*, **71**, 239
11. Fan, S.T., Yan, K.W., Lau, W.Y. *et al.* (1986) Tuberculosis of the pancreas: a rare cause of massive gastrointestinal bleeding. *Br.J.Surg.*, **73**, 373
12. de Miguel, F., Beltran, J., Sabas, J.A. *et al.* (1985) Tuberculous pancreatic abscess. *Br.J.Surg.*, **72**, 438
13. Aggarwal, P., Wali, J.P., Jain, R. and Berry, M. (1989) Tubercular abscess of the pancreas. *Am.J.Gastroenterol.*, **84**, 985–986
14. Crook, L.D. and Johnson, F.P. Jr. (1988) Tuberculosis of the pancreas: a case report. *Tubercle*, **69**, 148–151

*(Accepted by S. Bengmark 24 October 1991)*

### INVITED COMMENTARY

This is a useful and interesting single case report of a benign pancreatic mass misdiagnosed as carcinoma resulting in pancreatico-duodenectomy. Fortunately, the combination of surgery and the appropriate anti-tuberculous medical treatment appears to have resulted in a good outcome.

Few conclusions can be drawn about the entity of pancreatic tuberculosis itself on the basis of such a case report other than to maintain awareness of the condition amongst HPB surgeons, particularly those working in areas where TB is endemic.

Had this case been set in a major hospital in the western world the central cavity in the pancreatic mass would almost certainly have been seen on CAT. Needle biopsy would most likely have been done, hopefully resulting in a different pre-operastive diagnosis and a different set of therapy decisions being made. Pancreaticoduodenectomy is not undertaken lightly. Though the outcome here was satisfactory we should remember that the useful limits of the cheaper technologies such as ultrasound should be fully explored. Repeat examinations are often useful where the initial examination has been suboptimal. Where limitations are placed by the presence of gas in the stomach and duodenum, this can on occasions be minimised by draining the stomach and then repeating the examination after filling the duodenum with water or fruit juice free of particulate matter. This is not a high technology exercise and in this case may have resulted in a more accurate pre-operative diagnosis had the pancreatic mass been adequately visualised by ultra-sound scanning. The cavity in the pancreatic mass may well have been detected and then biopsied percutaneously.

Whenever a pancreatic mass is being dealt with we must assume it is malignant until proven otherwise. Pancreaticoduodenectomy is not a cheap or risk free option for the investigation and diagnosis of pancreatic disease. Where diagnostic difficulties are encountered pancreaticoduodenectomy should only be resorted to when available non-operative diagnostic methods have been fully utilised and the nature of the underlying disease process remains in doubt.

Noel Tait
Department of Surgery
Westmead Hospital
Westmead, NSW 2145
Australia

### INVITED COMMENTARY

Gastrointestinal tuberculosis is not uncommon is developing countries but pancreatic involvement is rare. Tuberculosis of the pancreas can present as part of miliary tuberculosis, or as a secondary tuberculous focus with focal pancreatic involvement. This latter form is much rarer[1].

The diagnosis of tuberculosis of the pancreas is extremely difficult due to its rarity and its atypical presentations[1-4]. Haematological and immunological (Heaf, Mantoux) tests may show non-specific changes only[5]. Evidence of tuberculosis (active or healed) on chest X-ray may give a hint but such X-ray findings are common in areas where tuberculosis is prevalent. Radiographically demonstrable pancreatic calcification has been reported but such calcification occurs commonly in patient with chronic pancreatitis. It has been suggested that in a locality where chronic pancreatitis is rare and tuberculosis is prevalent such as in Hong Kong, pancreatic calcification should raise the suspicion of tuberculosis of the pancreas[2]. Abdominal ultrasonography, selective visceral angiography and computed tomography only show a non-specific pancreatic mass or abscess.

A high index of suspicion is mandatory to make a correct clinical diagnosis. Diagnosis is extremely difficult in patients with focal pancreatic involvement. In patients with miliary tuberculosis, the presence of a pancreatic mass may lead to a diagnosis of tuberculosis of the pancreas. Although diagnosis has been reported with ultrasound guided percutaneous aspiration from pancreatic abscess[3], and demonstration of acid fast bacilli in the pus, it is more commonly established postoperatively when the excised specimens came back as pathological surprises[1], or at post mortem examinations[2,4].

Treatment is straight-forward after a diagnosis is made. The mainstay of treatment is anti-tuberculous treatment. Surgery only served as a secondary role in draining abscesses and bypassing obstructed systems.

W.Y. Lau
A.K.C. Li
Department of Surgery
The Chinese University of Hong Kong
Prince of Wales Hospital
Shatin, New Territories
Hong Kong

### *References*

1. Crowson, M.C., Perry, M. and Burden, E. (1984) Tuberculosis of the pancreas: a rare cause of obstructive jaundice. *Br. J. Surg.*, **71**, 239
2. Fan, S.T., Yan, K.W., Lau, W.Y. and Wong, K.K. (1986) Tuberculosis of the pancreas: a rare cause of massive gastrointestinal bleeding. *Br. J. Surg.*, **73**, 373
3. Aggarwal, P. and Jain, R. (1989) Tubercular abscess of the pancreas. *Am. J. Gastroenterol.*, **84**, 985–986
4. Rushing, J.L., Hanna, C.L. and Selecky, P.A. (1978) Pancreatitis as the presenting manifestation of miliary tuberculosis. *West J. Med.*, **129**, 432–436
5. Kapoor, V.K. and Sharma, L.K. (1988) Abdominal tuberculosis. *Br. J. Surg.*, **75**, 2–3

